# Investigating visual navigation using spiking neural network models of the insect mushroom bodies

**DOI:** 10.3389/fphys.2024.1379977

**Published:** 2024-05-22

**Authors:** Oluwaseyi Oladipupo Jesusanmi, Amany Azevedo Amin, Norbert Domcsek, James C. Knight, Andrew Philippides, Thomas Nowotny, Paul Graham

**Affiliations:** ^1^ Sussex Neuroscience, School of Life Sciences, University of Sussex, Brighton, United Kingdom; ^2^ Sussex AI, School of Engineering and Informatics, University of Sussex, Brighton, United Kingdom

**Keywords:** mushroom body, insect navigation, spiking neural networks, visual learning, biorobotics, computational neuroscience

## Abstract

Ants are capable of learning long visually guided foraging routes with limited neural resources. The visual scene memory needed for this behaviour is mediated by the mushroom bodies; an insect brain region important for learning and memory. In a visual navigation context, the mushroom bodies are theorised to act as familiarity detectors, guiding ants to views that are similar to those previously learned when first travelling along a foraging route. Evidence from behavioural experiments, computational studies and brain lesions all support this idea. Here we further investigate the role of mushroom bodies in visual navigation with a spiking neural network model learning complex natural scenes. By implementing these networks in GeNN–a library for building GPU accelerated spiking neural networks–we were able to test these models offline on an image database representing navigation through a complex outdoor natural environment, and also online embodied on a robot. The mushroom body model successfully learnt a large series of visual scenes (400 scenes corresponding to a 27 m route) and used these memories to choose accurate heading directions during route recapitulation in both complex environments. Through analysing our model’s Kenyon cell (KC) activity, we were able to demonstrate that KC activity is directly related to the respective novelty of input images. Through conducting a parameter search we found that there is a non-linear dependence between optimal KC to visual projection neuron (VPN) connection sparsity and the length of time the model is presented with an image stimulus. The parameter search also showed training the model on lower proportions of a route generally produced better accuracy when testing on the entire route. We embodied the mushroom body model and comparator visual navigation algorithms on a Quanser Q-car robot with all processing running on an Nvidia Jetson TX2. On a 6.5 m route, the mushroom body model had a mean distance to training route (error) of 0.144 ± 0.088 m over 5 trials, which was performance comparable to standard visual-only navigation algorithms. Thus, we have demonstrated that a biologically plausible model of the ant mushroom body can navigate complex environments both in simulation and the real world. Understanding the neural basis of this behaviour will provide insight into how neural circuits are tuned to rapidly learn behaviourally relevant information from complex environments and provide inspiration for creating bio-mimetic computer/robotic systems that can learn rapidly with low energy requirements.

## 1 Introduction

The foragers of social insect species, such as ants, are amazing navigators. Their limited neural resources (Chittka and Niven, 2009; Webb and Wystrach, 2016) are finely tuned to produce impressive performance when navigating through complex environments (Knaden and Graham, 2016; Buehlmann et al., 2020). Investigating the neural basis of ant navigational capabilities helps advance understanding of how information from the environment can be processed and stored efficiently in small neural circuits. A common challenge in the field of computational neuroethology (Datta et al., 2019) is translating the natural behaviours in question into experiments where variables can be modulated in a controlled manner. However, because forager ants are naturally motivated to find food and return to the nest, it is possible to craft navigation experiments that exploit this foraging behaviour to investigate how ants use visual cues when navigating (Vermehren et al., 2020; Zeil, 2022). The two key features of solitary ant navigation are path integration (PI) and visual route following (Wehner and Räber, 1979; Collett et al., 2013; Sun et al., 2020). PI in ants includes the ability to return home from a novel location by keeping track of their own outgoing movements. PI has two main requirements, a mechanism to keep track of speed/distance travelled such as odometry (Wittlinger et al., 2007; Wolf, 2011) and a mechanism to keep track of orientation using information derived from environmental compass cues and neuronal systems that track rotations (Wehner, 1976; Rössler, 2023). PI allows for ants to explore unfamiliar areas and return on direct paths to their nest when they have food. Furthermore the remembered PI coordinates of the successful foraging location can be used to guide a subsequent foraging route (Collett et al., 1999; Wehner et al., 2004). These PI guided routes to and from food and the nest can act as a scaffold (Collett et al., 2003) for rapid visual route learning (Mangan and Webb, 2012; Stone et al., 2017; Haalck et al., 2023). In this paper, we investigate whether providing images taken from a previous route can scaffold on-line visual route learning and navigation when a model of the ant mushroom body (MB) is used as the learning mechanism.

Visual navigation in insects is well-explained by view based navigation models (Zeil, 2012) inspired by the seminal “snapshot model” (Cartwright and Collett, 1983). In this type of model, memories of important locations are stored as snapshots or views. Therefore, to navigate to a goal, the ant only needs to move so as to make their current view similar to a previously stored snapshot taken at the goal location (Wehner and Räber, 1979; Nicholson et al., 1999; Durier et al., 2003). To extend this model to visual route guidance, a model has been proposed in which an ant compares its currently experienced view with a set of stored views, taken along the route (Philippides et al., 2011; Baddeley et al., 2012), and moves in whichever direction the current view best matches the stored snapshots. This mechanism allows ants to navigate along routes by sampling the world (Wystrach et al., 2014) and moving in directions that are characterised by the most familiar visual scenes. Lesion studies focusing on this route-following behaviour were able to show that learned visual navigation is mediated by the insect brain region known as the mushroom body (Buehlmann et al., 2020; Kamhi et al., 2020). Lesions made to the mushroom body impair an ant’s ability to remember visually guided routes, while keeping other innate navigation behaviours intact. Furthermore, modelling studies have also shown that the mushroom body could act like a visual familiarity detector, storing visual scenes and outputting proxies for familiarity when presented with new visual scenes (Ardin et al., 2016; Zhu et al., 2021). Given this evidence, we wanted to inestigate whether a spiking MB model could provide the same function in complex natural scenes.

**FIGURE 1:**
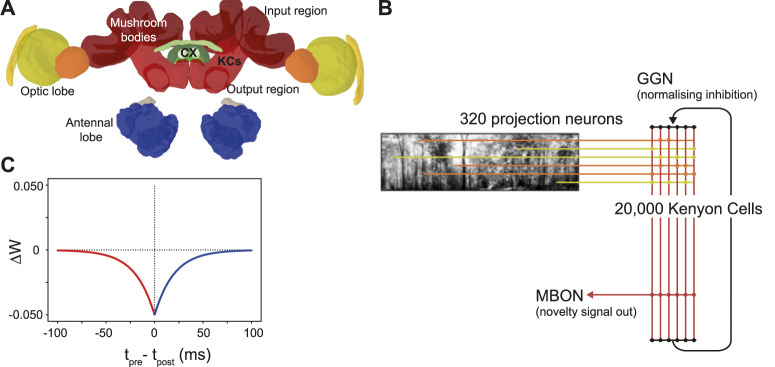
The ant mushroom body and mushroom body model structure. **(A)** Diagram of *Cataglyphis nodus* brain (Habenstein et al., 2020). Adapted from Insect brain database under CC BY 4.0 License. Mushroom bodies shown in red, medulla shown in yellow, lobula shown in orange. The anatomical position of inputs, outputs and Kenyon cells (KCs) are labelled as well as the central complex (CX) which is one of the major output regions. **(B)** Mushroom body model structure. Pixel intensity of input images are converted into an input current for visual projection neurons (VPNs). VPNs are sparsely connected to the KC population via excitatory connections (horizontal lines). All KCs are connected to mushroom bodies via an excitatory connection with an anti-hebbian STDP learning rule. Learning via this learning rule is switched on during model training and switched off during model testing. IFN - inhibitory feedback neuron, MBON–mushroom body output neuron. **(C)** The learning rule for the anti-hebbian spike time dependent plasiticity. *T*_pre_–spike time of presynaptic neuron, *T*_post_–spike time of post synaptic neuron. Δ*W*–Size of the weight change. Red - Presynaptic neuron fires before postsynaptic neuron. Blue–Presynaptic neuron fires after postsynatic neuron.

The mushroom bodies are prominent bilateral brain regions located to the left and right of the insect brain midline. Each mushroom body is composed of a calyx that receives input from other (mainly sensory) regions, a peduncle containing a large population of intrinsic neurons known as kenyon cells (KCs), and vertical and medial lobes containing mushroom body output neurons (Aso et al., 2014). The mushroom bodies of insects (particularly *Drosophila*) are well studied for their role in olfactory learning (Busto et al., 2010). However, in addition to olfactory inputs, *ant* mushroom bodies have significant inputs from primary visual areas. The MB collar region has connections from the medulla and lobula in the optic lobe (Figure 1); receiving ipsilateral connections via the optical calycal tract (OCT), and receiving both contralateral and ipsilateral connections to both MBs via the anterior superior optic tract (ASOT) (Habenstein et al., 2020). The vertical lobe–one of the output regions of the MB–has a feedback connection to the MB calyx, likely helping to regulate KC activity (Habenstein et al., 2020). The *Drosophila* MB makes downstream projections to areas implicated in steering or motor control such as the lateral accessory lobe (LAL) and the central complex (Li et al., 2020; Steinbeck et al., 2020), and while there is limited evidence for this specifically in the ant, it is plausible that these could be downstream areas for the ant MB to act on to drive navigation behaviours.

Since neuronal recordings are difficult to obtain in navigating insects, computational neuroscience and robotics have been integral in bridging the gap between observed insect navigation behaviours and theories of neural computation (Mangan et al., 2023) not least as we have such good knowledge of the connectivity detailed above. In this neuroethological approach, theories of what strategies or neural architecture insects use to navigate are embodied in simulation or physically on robots. It is a powerful approach that focuses on producing models directly relevant to the behaviour of animals, but comes with limitations when falsifying or accepting concrete hypotheses (Webb, 2006; 2020). In this exciting field, a range of models have started to address the question of how mushroom bodies are used for visual navigation. Some take an algorithmic approach, where they look at the theorised processing that occurs in the mushroom body, and use analogues found in computer vision techniques to try and recreate navigation behaviours by embodying models in robotic or simulated agents (Möel and Wystrach, 2020; Stankiewicz and Webb, 2021). Another approach has been to create (rate-based) artificial neural networks (ANNs) which follow the general neural architecture of the MB and embody this in a robot vehicle or agent based simulation (Gattaux et al., 2023; Yihe et al., 2023), or utilise it as part of a larger navigation system simulating other navigation-related brain areas (Sun et al., 2020; Goulard et al., 2023). Finally some use spiking neural networks (SNNs) to create navigation models that mimic both the architecture and neuronal dynamics of the MB (Ardin et al., 2016; Müller et al., 2018; Zhu et al., 2021), or use SNNs as one component of a hybrid SNN/algorithmic navigation system (Nowak and Stewart, 2019).

Building on these computational analyses of mushroom bodies (MBs), we made an SNN MB model to increase our understanding of how the MB architecture learns visual information for navigation. While previous studies have shown examples of MB-inspired algorithms in various contexts, our goal is to shed light on how the dynamics of KC activity directly contribute to learning visual input by comparing changes in KC activity to the changes of incoming visual stimulus. In addition, we investigate the importance of model parameters such as connectivity sparsity, stimulus exposure time and the number of stimuli the model was trained on to the overall performance of the model. Finally, we test whether the insights gained from these investigations hold in the real world, by embodying our SNN MB model on a robot and comparing its performance to standard visual navigation algorithms (Zeil, 2012; Knight et al., 2019; Husbands et al., 2021; Amin et al., 2023).

## 2 Materials and methods

We chose to model using spiking neural networks (SNNs) as they are more biological representative than standard artificial neural networks (ANNs), and because they more closely mimic the electrophysiological activity/dynamics of biological neurons than rate-based models (Tavanaei et al., 2019). We built the model using PyGeNN (Knight et al., 2021), a Python interface for GeNN (Yavuz et al., 2016), which is a library that accelerates spiking neuron simulations by automatically generating optimised NVIDIA CUDA code–enabling complex models to be run on GPUs. GeNN is fast and memory efficient, allowing large-scale spiking simulations of complex networks to run on consumer accessible hardware at speeds comparable to dedicated supercomputers or neuromorphic systems (Knight and Nowotny, 2020). For us, GeNN meant we were able to run a very large spiking model consisting of over 20,000 neurons on a portable on-board processor, specifically, the Nvidia Jetson TX2.

**FIGURE 2:**
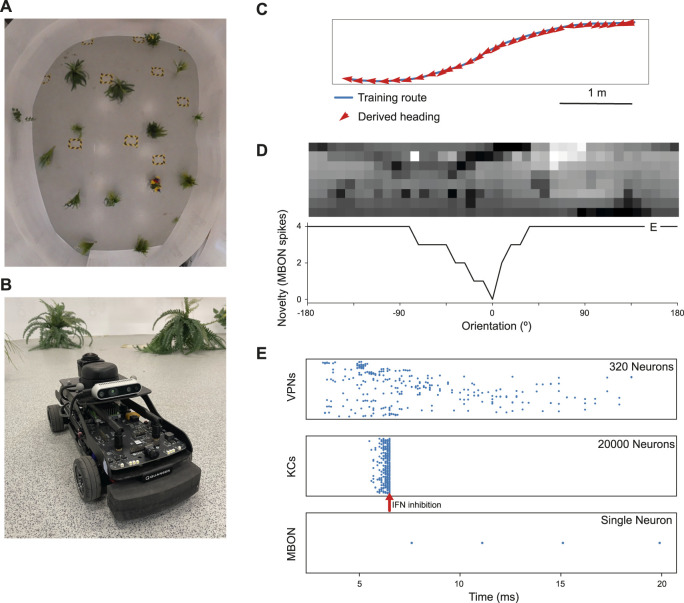
Model performance on a real world navigation task. **(A)** Top down view of arena used for robot tests and indoor image acquisition. **(B)** Image of the Q-car (Quanser) robot used for evaluating onboard navigation performance of the model **(C)** Model navigation behaviour on a typical training route (blue). Mean heading deviation = 10.7°. **(D)** Top - Example input panorama from the testing route that has been downscaled to 40 × 8 resolution. Bottom - Rotational response curve showing the MBON response at each orientation of the panorama. 0° is the original orientation of the route image and MBON activity (novelty) is lowest for the familiar, trained image. **(E)** marker shows the location for the image orientation used in **(E)**. **(E)** Raster plots showing spiking activity in each population of neurons. VPNs–visual projection neurons, KCs–Kenyon cells, MBON–mushroom body output neuron, IFN–Inhibitory feedback neuron. IFN threshold was set to 200mv for this experiment. Arrow–shows the time point where the IFN started to inhibit the KC population. The MBON activity indicates that the image is novel and not part of the training route.

Inspired by currently known neuroanatomy and by the structure of previous mushroom body models based on both ant navigation (Ardin et al., 2016) and *Drosophila* olfaction (Nowotny et al., 2005), we produced a model that includes simplified versions of key features of the mushroom body (Figure 1B). Our model however differs in our use of feedback inhibition, which better fits data observed from mushroom body structures in the locust (Kee et al., 2015) and *Drosophila* (Rapp and Nawrot, 2020). Another key difference in our model is the lack of a Dopaminergic neuron (DAN) for model simplification reasons. Since we were not investigating what factors induce learning, we were able to simplify the model by turning learning on during training and turn learning off during testing. Our model has four neuron types: visual projection neurons (VPNs), Kenyon cells (KCs), a mushroom body output neuron (MBON) and a single inhibitory feedback neuron (IFN) to regulate the activity of the KCs. Although the specific number of inhibitory neurons innervating the ant KCs is not known, to simplify our computations we represented inhibition with a single neuron innervating all KCs. Learning between the KCs and the MBON is turned on during training and turned off during testing the model. The VPNs receive visual input in the form of direct stimulation currents. For stimulus presentation, each pixel from an image is mapped to one VPN, and the brightness of the pixel directly determines the strength of stimulation to its VPN. Because the VPN-to-pixel ratio is 1:1, the VPN population count is dependent on the input image resolution. We tested at a resolution of 40 × 8, so our VPN population contained 320 neurons. The next population in the network is the KC population, consisting of 20,000 neurons. The synapses between the VPNs and KCs are excitatory and non-learning, and the connectivity is sparse, that is, each KC is connected to a low number of randomly chosen VPNs. The IFN is a single neuron, which provides feedback inhibition to the entire KC population. The final neuron type is an MBON, and the activity of this neuron is linked to behavioural outputs of the robot in our tests (see Figures 2D, E). The KCs are connected to the MBON with all-to-one excitatory connections that follow an anti-Hebbian spike-timing dependent plasticity (STDP) rule (Figure 1C) as described below. As discussed in a *Drosophila* olfaction modelling study (Bennett et al., 2021) and shown in an experimental study (Hige et al., 2015), there is evidence that the *Drosophila* KC to MBON connection is depressed after learning occurs. Although from a different sensory modality and species, we took inspiration this and, as with Ardin et al. (2016) we used a learning rule that utilises depression in an STDP synapse.

### 2.1 Neuron and synapse equations

In this work, VPNs, KCs and the MBON are modelled as Leaky Integrate-and-Fire (LIF) units where the membrane voltage *V*_*i*_ of neuron *i* is modelled as:

$$\tau_{\text{m}}\frac{dV_{i}}{dt}=\left(V_{\text{rest}}-V_{i}\right)+R_{\text{m}}\left(I_{{\text{syn}}_{i}}+I_{{\text{ext}}_{i}}\right),$$ (1)

where *τ*_m_ = 10 ms and *R*_m_ = 50 MΩ represent the time constant and resistance of the neuron’s cell membrane, *V*_rest_ = −60 mV defines the resting potential, $I_{{\text{syn}}_{i}}$ represents the synaptic input current and $I_{{\text{ext}}_{i}}$ represents an external input current used to deliver visual input to the VPN neurons.

When the membrane voltage crosses a threshold *V*_th_ = −50 mV a spike is emitted, the membrane voltage is reset to *V*_rest_ = −60 mV and updating of *V* is suspended for a refractory period *τ*_ref_ = 2 ms (see Table 1). The IFN neuron is modelled as a simple integrate-and-fire neuron.

$$\tau_{\text{m}}\frac{dV}{dt}=R_{\text{m}}I_{\text{syn}}$$ (2)

with membrane time constant *τ*_*m*_ and resistance *R*_*m*_ as above. The threshold on the IFN was set by a voltage parameter, where if the membrane potential reached that value it will fire and inhibit the KC population. The level of inhibition from the IFN to the KC populations was set very high (see Table 2) for the purposes of completely shutting off KC activity. Although those levels of inhibition are not biologically plausible, for the purposes of our modelling it gave us more direct control over the number of KCs firing.

**TABLE 1:** Parameters of Leaky-integrate-and-fire (LIF) neurons used in the model. LIF neurons were used for the VPN, KC and MBON neurons.

Parameter	Value
Time constant *τ*_m_	10 ms
Membrane resistance *R*_m_	50 MΩ
Resting potential *V*_rest_	−60 mV
Firing threshold *V*th	−50 mV
Refractory period *τ*_ref_	2 ms

**TABLE 2:** Synaptic parameters between different neuron populations. *τ*_syn_ is a time constant that affects the speed of the change of synaptic conductance. *w*_*ij*_ is the weight of the connection, and for the KC-MBON it is the starting weight. The KC-IFN connection does not have a *τ* value as the synapse uses a delta current where all current is transferred to the IFN in a single timestep.

Connection	*τ*_syn_ [ms]	*w*_*ij*_ [nA]
VPN-KC	3	0.25
KC-MBON	15	0.005
IFN-KC	5	−5
KC-IFN	N/A	1 [mV]

Synaptic connections are current-based with presynaptic spikes leading to exponentially-decaying input currents $I_{{\text{syn}}_{i}}$ ,

$$\tau_{\text{syn}}\frac{dI_{{\text{syn}}_{i}}}{dt}=-I_{{\text{syn}}_{i}}+\overset{n}{\underset{j=0}{\sum }}w_{ij}\underset{t_{j}}{\sum }\delta \left(t-t_{j}\right),$$ (3)

where *τ*_syn_ represents the synaptic time constant (for values see Table 2) and *t*_*j*_ are the arrival times of incoming spikes from *n* presynaptic neurons. The exception to this is the KC to IFN connection, where presynaptic KC spikes lead instantly to voltage jumps in the IFN. The ordinary differential Eqs 1, 3 are solved with an exponential Euler algorithm.

The VPN to KC connections were initialised with a set weight and set number of VPN connections per KC (value dependent on experiment; detailed in model evaluation methods), with the connectivity pattern being determined randomly. The KC to MBON synapses were initialised at a value of 0.005, with all KCs connected to the single MBON. The anti-Hebbian learning rule used on the synapses between the KC and MBON populations modifies the synaptic weight (*w*_*ij*_) between a pre and postsynaptic neuron based solely on the relative timing of pre (*t*_pre_) and postsynaptic (*t*_post_) spikes (Δ*t* = *t*_post_ − *t*_pre_) according to four

$$\Delta w_{ij}=-Ae^{-\frac{\left|\Delta t\right|}{\tau }}$$ (4)

where *A* = 0.05 nA represent the learning rate and *τ* = 2 ms represents the time constant of the STDP kernel. Values of *w*_*ij*_ are clamped between 0 and 0.05. This rule is used with a *nearest-neighbour* spike-pairing scheme in which only the pairs formed by a presynaptic spike in neuron *j* and the directly preceding postsynaptic spike in neuron *i* (and *vice versa*) are considered when updating *w*_*ij*_. This learning rule implies that when a KC and the MBON fire close together in time, the synapse connecting them weakens. As a result, when a KC is repeatedly activated in close time proximity to the MBON firing, that specific KC is less likely to stimulate the MBON in future trials. Synaptic learning occurs during the training phase. In this phase, each image from the training set or training route is presented in sequence for a set amount of ms (amount of time is dependant on experiment), during which the weights matrix between the KCs and MBON is updated according to the STDP rule. In the testing phase, learning is turned off so there are no weight matrix updates.

### 2.2 Model evaluation methods

In our first test on model heading selection accuracy based on images from an indoor arena, and for the tests where we compared KC activity and image novelty the parameters were as follows: Each KC was randomly connected to 10 VPNs, with a weight of 0.25 from the VPNs to the KCs. The learning rate in the KC to MBON connection was set to 0.05, the IFN threshold was 200, and the presentation time of the stimulus to the model was 20 ms. In the parameter search these values varied as they were the parameters being investigated for their effect on navigation performance. In all of our tests, membrane voltage values of all neurons were reset between each stimulus presentation. To analyse how KC activity relates to the input images and how overall navigation performance relates to model parameters, we used a dataset of panoramic (360°). Images captured outdoors in a wooded area from a wheeled robot. The 360° panoramas were captured on a Kodak Pixpro VR action camera and were stored as 226 × 76 images. Before use in the modelling experiments, they were down-scaled to 40 × 8 and converted to gray-scale and the pixel values were inverted so that the darkest pixels produce the highest values. Normalisation was applied to the values of each pixel by subtracting the mean pixel value of each image, then dividing that value by the standard deviation. The pixel values are multiplied by a scaling factor used to determine the amount of current that each VPN receives through a current source injection throughout the image presentation. Each panorama has a coordinate and a heading orientation associated with it, allowing them to be used to test navigation capability, and from this dataset we used 400 images. These 400 images were taken along a 27 m route, with an image being taken every 7 cm of travel (on average).

For each run of the parameter search, the route was separated into a separate training and testing set where alternating images were placed in the train or test set at a 50% split. During training the model is trained on the training set images in their original, correct heading direction. In a test the model is presented with 40 rotations of each input image from the test set rotated from 0 to 351° from the true heading in steps of 9°, mimicking the effect of an agent rotating to all the different headings. During the test process the MBON response is recorded. The heading of the test image that has the minimum MBON response is taken as the direction of travel that the model would choose when at that location (see Figures 2C, D). The performance metric is the mean heading deviation which is the mean of the absolute angular deviation between the heading given by the lowest MBON response and the true heading for each image in the test set. In the parameter search we modulated the training process using a “training proportion” parameter, which controlled how many images from the training set are used to train the model. When using this parameter the initial train and test split is unaffected. The training proportion and number of images in the initial training set is used to calculate number of images in a new training set. This number of images is selected from the original training set, with the images selected evenly spaced from the start to the end of the original training set. The new training set is then used to train the model. Regardless of the training proportion parameter, the model is tested on the entire test set.

### 2.3 Robot evaluation methods

To evaluate real world performance, the mushroom body model was embodied on a Q-car (Quanser) robot (Figure 2B). This is a 4-wheeled vehicle, measuring approximately 39*L* × 19*W* × 20*H*cm, with forward drive provided by a central motor and Ackermann steering provided through a pair of servo-motors attached to the front two wheels. Panoramic images were acquired by connecting a Kodak PixPro camera and all processing was performed on-board using an NVIDIA Jetson TX2.

The robot arena (Figure 2A) consists of a 5*m* × 4*m* indoor space, surrounded by white walls and populated with artificial plants approximately 30 cm wide. For accurate ground truth of the robot’s movements, we utilise a VICON motion capture system. Training routes are acquired through manual control of the robot, with images captured only while the robot is in motion. This is to prevent training on repeat images from instances where the robot is static. Once a navigation model is trained on these images, test trials are carried out by returning the robot to the starting position and then starting navigation (process described below). For each of the models considered, a total of 5 trials were conducted. In this work, two routes spanning the length of the arena were considered. A simple 6.5 m route consisting of a straight portion followed by a single turn and a more complex 7.1 m ‘snaking’ route consisting of both a right and left turn, where there is a more dramatic change in the views perceived by the robot.

In addition to the mushroom body model, two standard view based navigation models were implemented for comparison at the same resolution of 40 × 8 pixels: In the *Perfect Memory* model, the views perceived by the agent during the first traversal are directly stored in memory. During recapitulation, the agent makes a pixel-wise comparison of every rotation of the current view with every snapshot, choosing the heading based on the rotation of the current view which results in the smallest of these differences (Zeil, 2012; Knight et al., 2019; Husbands et al., 2021). The robots driving speed was the same, with variance in how long the trials take between models resulting from model inference speed. In the *Infomax* model, a single layer neural network is trained on the snapshots using the Infomax (Bell and Sejnowski, 1995) learning rule, after which these images are discarded. This learning rule adjusts weights such that the information provided by the output units about the input unit activity (via mutual information) is maximised. In this way, the network encodes a holistic representation of the route and can be used to recall familiarity when presented with a view. There are 320 input neurons, corresponding to each pixel, and the network is fully connected to 320 novelty neurons. In a similar process, each rotation of a view is presented to the network for a familiarity value, with the orientation corresponding to the greatest familiarity being used to set the robot’s heading. In work by (Husbands et al., 2021), both the Perfect Memory and Infomax models demonstrated successful navigation of the same outdoor 60 m route on board a robot (Husbands et al., 2021). The additional models implemented on the Q-car replicate this work, as well as drawing on recent refinements to the Infomax variant which improve the robustness of navigation (Amin et al., 2023).

## 3 Results and implications

We first tested the model on a navigation route represented by a series of panoramic images collected in our indoor robot testing arena. We trained it on 68 images from points along the route (Figure 2C) and then tested its ability to choose the correct heading direction. Each panoramic image was presented to the model for 20 ms of simulation time, and our IFN was set to inhibit the KC population after 200 KC neurons had fired. On this route, the model performance was good, with a mean heading deviation across the route of 11° (Figure 2C). Thus we have an existence proof that the model can learn a representation of the views needed to guide the route.

**FIGURE 3:**
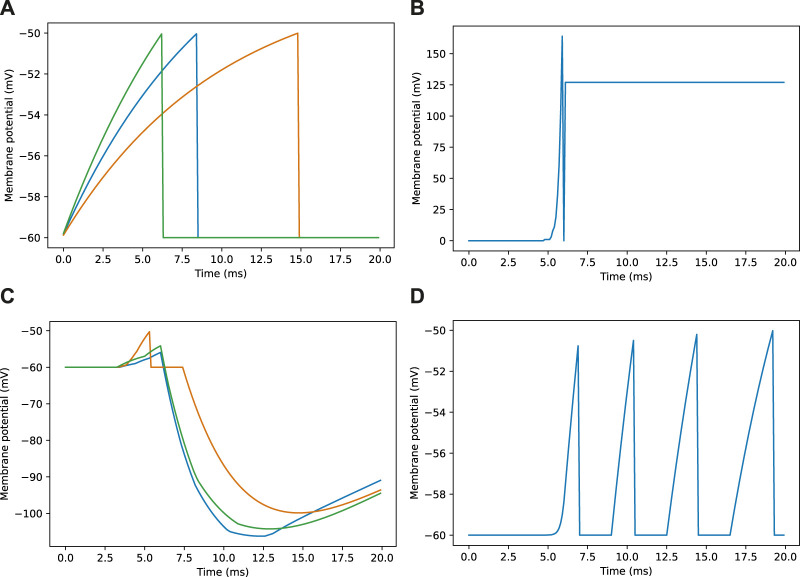
Example membrane potential traces for neurons during a navigation task. Traces are taken from a sample of neurons responding to an image as seen in Figure 2E. **(A)** Membrane potential traces for three visual projection neurons (VPNs). **(B)** Membrane potential for inhibitory feedback neuron (IFN). **(C)** Membrane potential traces for three kenyon cell neurons (KCs). **(D)** Membrane potential for mushroom body output neuron (MBON).

Looking closer at the neuronal firing during these trials (Figures 2E, 3), we see the typical dynamics of neuronal responses to a route view. The VPNs that were linked to the highest pixel intensity neurons start to spike at around 2 ms with most VPN spikes taking place between 2 and 10 ms of presentation time, after which the spikes began to taper off (Figure 2E top). The VPN spikes at this later stage are either reoccurring spikes linked to high intensity pixels which spike again after their refractory period has finished, or spikes from VPNs linked to medium intensity pixels that took longer to gain sufficient input current to produce a spike. In 3A there are three examples of VPNs which spike at different times, due to the pixel that they receive input from giving varying amounts of stimulation. The KC spiking is heavily concentrated around the 5 and 6 ms mark as sufficient time is needed to gain input from connected VPNs (Figure 2E middle). The KC spiking stops abruptly at close to 6 ms (see 3B, where the extremely strong inhibitory current from the IFN neuron stops any KC activity. It should be noted that although the IFN was set to fire after receiving stimulation from 200 KC spikes, a small amount more than 200 KCs fire in the duration of a single trial. This is because of the time constant attached to the IFN-KC synapse (see Table 2), which means that after the IFN theshold is reached there is a short interval of time where a small number of additional KCs can fire before all they are completely inhibited. The additional KC spikes cause some further depolarisation of the IFN after it spikes (see Figure 3B). Figures 2E, 3D show the response to an image of maximum unfamiliarity where the MBON spikes 4 times. Note that the extended firing is enabled by the 15 ms time constant of the KC to MBON synapses so that spiking continues for a while even after KC activity has stopped. Figure 2D highlights the functional property of the MBON firing in a navigation context. Because KC to MBON synapses are depressed when learning the route, familiar scenes produce low MBON firing rates and novel scenes, as in the example shown in Figure 2E, produce more spikes.

### 3.1 Kenyon cell activity and image novelty

**FIGURE 4:**
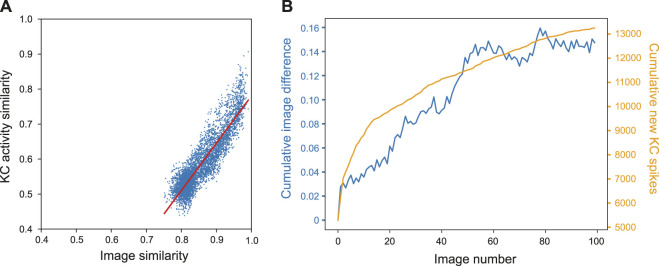
Influence of image novelty on KC activity. **(A)** Cosine similarity of image pairs compared to cosine similarity of KC activity in response to those images. Pearson correlation R score = 0.901, *p*-value = 0.000, gradient = 1.352. **(B)** Cumulative image difference along the route dataset, compared to the cumulative number of new KCs firing for each image along the route.

To demonstrate how the property of familiarity detection emerges out of the MB architecture we analysed the KC activity in relation to the difference between input images. We used a set of 400 woodland images captured on a robot. Each image was presented to an untrained model for 20 ms while the KC activity was recorded. For each possible pair of images we calculated the cosine similarity by converting images into a vector of values where each value corresponds to the intensity value of the respective pixel. Similarly, we calculated the similarity between pairs of KC spike trains by converting the spike trains into binary vectors where each 1 or 0 represents whether a specific KC was active or not in response to an single image presentation. Figure 4A shows that the correlation between the measured distances was strong; the Pearson correlation test R score was 0.901, *p*-value = 0.000, line of best fit gradient = 1.352.

This shows that, despite VPN to KC connectivity being random, KC activity is representative of the differences in image space–an important condition for familiarity discrimination. However, although the correlation is strong, the grouping of the results indicates that KC activity does not correspond to image similarity in a 1:1 fashion. Most results are found in the range of 0.5–0.6 for the KCs and 0.77–0.84 for the images (median of 0.56 and 0.84 respectively). At this point of relatively high similarity, the KC activity similarity is much lower, indicating that even when images are very similar, the network responses enhance the differences between inputs.

We further investigated how KC activity is related to input novelty by comparing cumulative image difference with the cumulative number of new KCs firing during the model training process; if related, the tallies should follow similar trends. We trained the MB model on a sequence of 400 images from the outdoor wood dataset (Methods). For each image presented to the model we recorded the identity of each kC cell that fired, and if a KC fired that had not fired previously during training, it was added to the cumulative total. To calculate the cumulative image difference, we calculated the structural similarity between each image and all the previously presented images using the metrics.structural_similarity function in scikit-image (van der Walt et al., 2014). The resulting similarity scores were then averaged and subtracted from one to represent image difference rather than similarity. While the image difference algorithm is not exactly how a biological system will differentiate between images, we tested this on the basis that if the KCs are encoding novelty, then new KC spikes (KCs that have not spiked up to that point during training) will be related to input image novelty. Figure 4B shows that the variables clearly follow the same general trend, indicating that when new KCs spike, the network is detecting novelty and will be able to learn that new stimulus (because of the independence of the new KC code, relative to previously learned inputs). For this particular set of input images, there is eventually a plateau in new KC spikes and cumulative image difference. This may reflect the ‘capacity’ of the network or the inherent similarity in the route images used.

### 3.2 Effect of parameters on model performance

**FIGURE 5:**
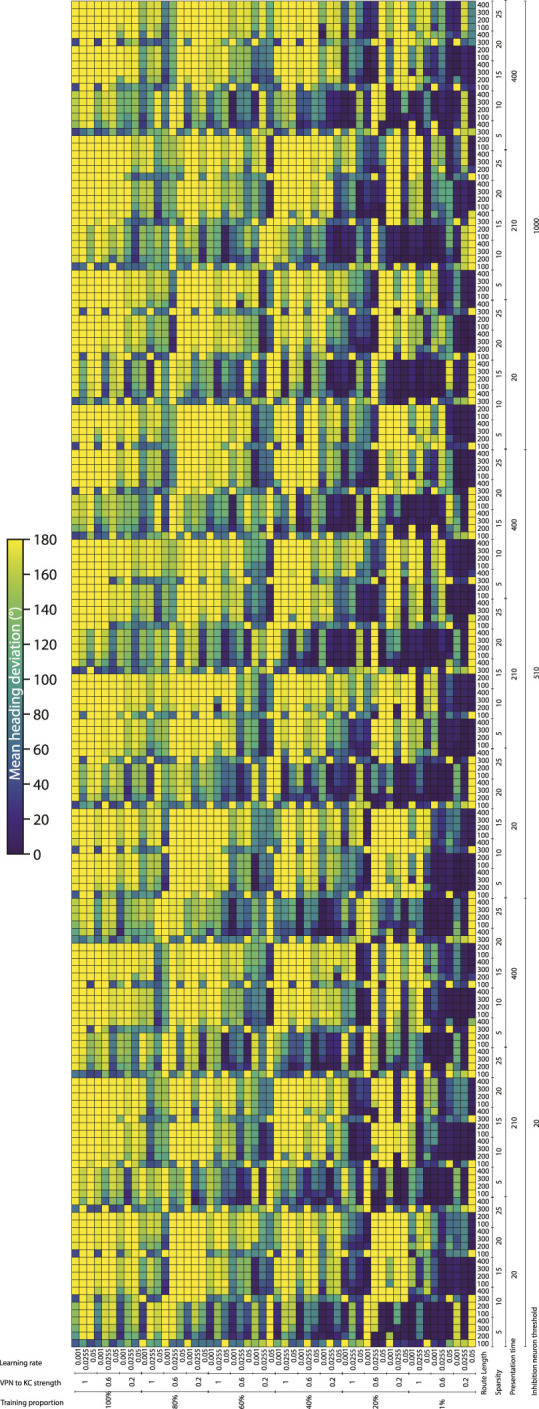
Navigation performance and confidence across different model parameters. Each cell is a single run of the model with the performance metric show via color. Navigation accuracy represented via the mean heading deviation metric shown via the colour bar, where lower values and darker colours correspond to better performance. 9,720 runs of the model were carried out in total. (**Horizontal axis**) The training proportion value changes every 9 cells, changing between the values 1%, 20%, 40%, 60%, 80% 100%. The VPN to KC strength value changes every three cells, cycling through the values 0.2, 0.6, 1 (nA). Learning rate changes every cell, cycling through the values 0.05, 0.0255, 0.001 (au). (**Vertical axis**) IFN threshold changes every 60 cells, changing between the values 20, 510, 1,000 (mV). Presentation time changes every 20 cells, cycling between the values 20, 210, 400 (ms). VPN connections per KC changes every 4 cells, cycling between the values 5, 10, 15, 20, 25. Route length changes every cell, cycling between the values 100, 200, 300, 400.

To systematically evaluate how various simulation parameters affect the performance of the MB model, we conducted a grid search (see Figure 5 for full search results). We tested 7 parameters in total (see Table 3): route length (as a way of testing task difficulty), stimulus presentation time, training proportion (amount of the route used to train the model), connection sparsity between the KCs and the VPNs, IFN threshold (used to control the amount of KCs firing), learning rate and connection strength between the VPNs and KCs. We chose parameter values by linearly spacing parameters between two bounds, and in total we tested 9,720 configurations of the model completing the navigation task. The two metrics we analysed from each run was mean heading deviation and confidence. Mean heading deviation averages the model’s absolute angular deviation from the true heading direction during testing. During testing, the angle of rotation that receives the minimum response of the MBON is used to give the heading of the model (Figure 2D). However, sometimes multiple angles receive the minimum response from the MBON, therefore we calculated confidence as one minus the number of angles that had the minimal response divided by the total number of rotations. Thus a score of one is maximum confidence where the MBON had a minimum response to a single angle, while a score of 0 is the lowest confidence as the MBON responded to every angle available. Although some extreme values were chosen in the search, 15% of mean heading deviation results were 20° or below, showing there are a large amount of model configurations we tested that are capable of accurate navigation. Below we present notable results and parameter interactions from the grid search, isolating parameters by averaging across the results at specific parameter values. For all results of every run see Figure 5.

**TABLE 3:** Values tested in parameter search.

Parameter name	Values	Description
Training proportion (%)	1, 20, 40, 60, 80, 100	Amount of a route the model is trained on
VPN to KC strength (nA)	0.2, 0.6, 1	Strength of excitation from VPN to KC neurons
Learning rate (au)	0.05, 0.0255, 0.001	Scales the extend of the weight change within the learning rule (higher value results in larger change)
IFN threshold (mV)	20, 510, 1,000	Number of KC inputs the IFN receives before it spikes
Presentation time (ms)	20, 210, 400	How many milliseconds the model is presented with each stimulus
Sparsity (au)	5, 10, 15, 20, 25	Number of VPNs each KC is randomly connected to
Route length (au)	100, 200, 300, 400	Number of images the model is tested on (higher route length is a longer distance)

Connection sparsity had a clear effect on results, with a value of 5 (each KC connected to 5 VPNs) providing the best results overall when all other parameters were averaged. However, how connection sparsity affected performance was highly dependent on other parameters. For example, at a presentation time of 210 and 400 ms, the best number of KC to VPNS connections was 5, but at a presentation time of 20 ms the best performing sparsity level was 10. An explanation for this is that slightly less sparse connectivity helps counter extremeley low presentation time by making it more likely that spikes in the VPN population excite a sufficient amount of KCs. These parameters are interdependent because they both affect the amount of information allowed through to the KC population. Increased number of connections from VPNs to KCs means information from a wider number of pixels gets to the KCs, and increased presentation time means that VPNs that normally would not have enough time to cross their activation threshold and spike, may have enough time to spike and contribute to the activation of KCs. Averaging across all runs and isolating the presentation time parameter, presentation time did not have a linear effect on performance. The values 20 and 400 ms both had the mean heading deviations of 125°, while 210 ms had a mean heading deviation of 104°.

The IFN threshold had a minimal effect on the model’s performance when averaged across all other parameters, with mean heading deviation decreasing from 126° at a threshold of 20, to 116° at a threshold of 1,000. The learning rate had a large effect on mean heading direction, from 132° at a learning rate of 0.05, to 93° at a learning rate of 0.001. A key point of failure in the model occurs when the model is “saturated”, where too many KC to MBON connections are weakened, so that when the model receives any stimulus the MBON does not fire causing the stimulus to be marked as familiar. Provided other parameters are set high so that enough excitation reaches the KCs for learning to occur, a low learning rate allows the KC to MBON connections to be weakened to a lower extent, meaning that this type of saturation failure is less likely. The best VPN to KC connection strength in the parameter search on average was 0.2. However this interacted with the IFN inhibition threshold in a non linear fashion, where 0.6 was the optimal VPN to KC strength when the IFN inhibition threshold was set to 510.

Training proportion presents a general trend from the values of 100%–21%, where the less the model is trained the better it performs. However, there is a threshold where there is insufficient learning to drive good navigation performance, and this threshold is different depending on other parameters. At higher percentage connectivity levels and higher route lengths the model is more robust to very small amounts of training. In contrast, at shorter presentation times and shorter overall route lengths the model is less robust to very small amounts of training. At the best overall performing connection sparsity levels of 5, the best performing amount of training was using 21% of the route. When the amount of training is reduced even further to just 1% of the route, the training is insufficient and the model’s accuracy decreases. This effect is especially prominent at the presentation time of 20 ms, where the combination of low stimulus exposure time and low training amount of 1% causes a drastic decrease in performance compared to the same presentation time but with training using 15% of the route. This behaviour of less training data being better is in contrast with other visual navigation algorithms such as Infomax where more training improves performance consistently (Amin et al., 2023).

The route length parameter was used as a way to set the task difficulty, and our results follow this as increased route lengths have lower performance across a variety of parameters. The major exceptions to this are where the training amount is set to 1% (Figure 5). In these cases the higher route lengths are the only runs where training on 1% of the route gives the model enough exposure to multiple parts of the route. Looking at the most difficult route (400 images), we can see that the network can still produce good performance. However the range of parameters over which good performance can be produced is much narrower than for other route lengths. The parameters for the best performing run (12° mean heading deviation) at a route length of 400 were: training proportions of 40%, connection sparsity of 5, presentation time of 20 ms, IFN threshold of 20, learning rate of 0.001, and a VPN to KC strength of 0.6. This indicates that for this specific model the best way to learn longer routes is to learn a medium proportion of the route, with a low amount of exposure to aid in avoiding “saturation” of the model where too many KC to MBON weights are decreased.

As with heading deviation, the confidence scores across an entire run are averaged. The results from the model confidence followed the mean heading deviation very closely, where runs that had high accuracy results (low mean heading deviation) also had high levels of confidence. The main exception to this when the training proportion was at 1%, where even if for the same parameters there was very low performance, the confidence of the model was often very high.

### 3.3 Robot performance evaluation

**FIGURE 6:**
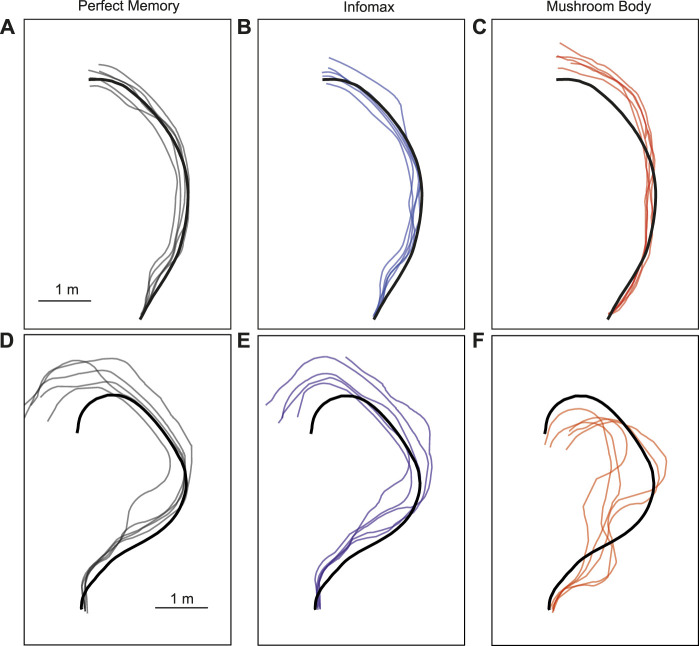
Example trajectories of MB and comparator models. The mushroom body model **(C, F)** is compared to Perfect memory **(A, D)** and Infomax **(B, E)** algorithms for two routes of differing sinuosity **(A–C, D–F)** in the robot arena. The thick black line is the training route and thin lines represent route recapitulations.

The parameter search provided a range of parameter values which produced good navigation performance in simulation. We therefore used these as a starting point for testing the mushroom body model in robot experiments. We trained and tested the model on two routes, and for comparison tested the same routes with the perfect memory (Zeil, 2012; Knight et al., 2019) and Infomax (Bell and Sejnowski, 1995; Amin et al., 2023) variants of the navigation algorithm. Each model had 5 trials per route, and to evaluate performance we used the mean distance from each point on the training route to the nearest point on the trial route. We used an image resolution of 40 × 8, with 20 rotations corresponding to a range of −90 to +90° from the current orientation. This means there is an up to ±4.5 error on the directed heading at each step, so in some cases this may contribute to divergence from the training path. On the simpler route which involved a gentle curve, all models performed comparably in terms of the mean distance to training route in metres: Perfect memory - 0.115 ± 0.057, Infomax - 0.112 ± 0.083, Mushroom body - 0.144 ± 0.088 (Figure 6; Table 4). Compared to the other models, the mushroom body had a tendency to overshoot the end point of the route. On the more sinuous route, the mushroom body performed worse than the other models: Perfect memory - 0.239 ± 0.04, Infomax - 0.27 ± 0.037, Mushroom body - 0.363 ± 0.02 (see Figure 6; Table 4); and also undershot the training end point. Although the robot’s speed when moving forward was consistent between trials, the MB model executes much slower than infomax and perfect memory (about 50 times slower) meaning that the robot travels for longer before executing the next move, which means it travels for longer on a fixed arc. This causes both overshooting in the simple curve case (Figure 6C), but also the excessive curved trajectories in the complex route (Figure 6F). Since during the test Infomax/Perfect memory compute much faster, they are able to do more interrogations of the input images, meaning they have more opportunity to correct their course.

**TABLE 4:** Performance metrics for robot tests. The simple route was a 6.5 m route with a gentle curve. The complex route was a 7.1 m route with multiple “snaking” curves. Metric shown is the mean distance of the robot from the original training route over 5 trials ±standard deviation.

Model	Simple routeerror [m]	Complex routeerror [m]
Perfect memory	0.115 ±0.057	0.239 ±0.04
Infomax	0.112 ±0.083	0.27 ±0.037
MB	0.144 ±0.088	0.363 ±0.02

## 4 Discussion

Our goal was to develop a spiking mushroom body model based on the known connectivity of the ant brain that can learn to navigate real world routes in order to investigate how neural activity and connectivity within the mushroom body allow effective learning. Inspired by previous work, we have built a model that can learn a set of images with their respective orientations and we have demonstrated how neural activity is converted into navigation behaviour. While it has been generally accepted in modelling studies that KCs encode novelty (Ardin et al., 2016; Müller et al., 2018; Sun et al., 2020; Zhu et al., 2021; Gattaux et al., 2023; Yihe et al., 2023) our results provided direct evidence that this mechanism can be used to solve real-world problems and we show how it comes about in the KC population. We have also demonstrated how the similarity of input images directly relates to overlapping neural activity in the KCs, and that the model encodes the novelty of input through the recruitment of newly firing KCs. Our parameter search helped us systematically analyse the effect of important features of the model, including how to avoid saturation in the KC to MBON connection.

The GGN in the locust and the anterior paired lateral (APL) neuron in the *Drosophila*, are large neurons that broadly innervate the mushroom bodies and provide inhibition (Liu and Davis, 2009; Papadopoulou et al., 2011; Kee et al., 2015; Rapp and Nawrot, 2020). Findings from the bee mushroom body in an olfactory learning experiment indicate that inhibitory feedback from GABAergic neurons onto KCs is an important aspect of learning (Boitard et al., 2015), and honeybee anatomy shows that there are multiple populations of neurons providing inhbitory feedback (Zwaka et al., 2018). Although it would be computationally more expensive, in future we could change our IFN implementation so that the IFN is constantly inhibiting KCs and changes its inhibition levels depending on the overall KC population activity. This may produce more granular changes in KC activity that we can use to investigate potential effects of feedback inhibition on model accuracy. To better match the anatomical findings of the honeybee which is more closely related to the ant as a fellow Hymenoptera, we could spread the inhibitory role to a wider population of neurons. This would also increase computational complexity, but it may be more biologically representative of ant brain dynamics. Another potential change we could incorporate is to take more inspiration from work done on variations to the standard Hebbian learning rule. There are many studies that apply varied versions of STDP to image/pattern recognition tasks (Vigneron and Martinet, 2020), and evaluating how these could apply to the mushroom body may improve performance of our model and grant new insights.

Previous mushroom body models set sparsity by having each KC receive approximately 10 connections (Ardin et al., 2016; Müller et al., 2018; Zhu et al., 2021) loosely based on the level of connectivity from sensory areas to the KC which has been estimated at 6 to 10 projection neuron connections to each KC (Turner et al., 2008; Li et al., 2020). Although there are caveats (these are approximations and are not from ants, and even if connections per KC is estimated, the overall sparsity will change depending on how many projection neurons are used to represent the visual stimulus in a model), through our parameter search, we found that overall connection sparsity levels within the ranges of 1.9–4.7 had good performance. With our VPN count of 320 neurons, this corresponds to each KC receiving 6 to 15 random connections each. However, we found that optimal sparsity of VPN to KC connections is highly dependent on other characteristics of the simulation, such as the amount of time the model is presented with each of the image stimuli. This provides important context for future modelling studies, as even if model parameters are informed by biology, the optimal value may differ greatly depending on other aspects of the simulation.

Through the parameter search, we have also shown that increased training does not necessarily correspond to increased accuracy, and the extent of this effect is different at different route lengths. We found the model can perform well by learning from a very small proportion of images spread out from the beginning to the end of a full route. Although previous studies have looked at the effect of different types of routes, they have not often focused on how the amount of training affects model performance, likely due to the intuition from more standard visual navigation models where more training means better performance (Baddeley et al., 2012; Amin et al., 2023). This characteristic of our model is related to the anti-Hebbian learning rule we are utilising during training and exposes details around the models information capacity. If in training the model is presented with too many images, too many kC cells will have had opportunity to spike in close time proximity to the MBON spiking, setting too many of the KC to MBON weights at or close to 0. When too many KC to MBON spikes are close to 0 this results in the MBON never spiking, which in output terms means every stimulus is classed as familiar, corresponding to bad navigation performance. In future, it will therefore be important to consider the amount of training to give a spiking model of the type we have presented depending on the route it will travel. This characteristic of learning being spread out at points along a route is reminiscent of how ants conduct learning walks in which learning is thought to be spread to key points of their outward trajectories (Nicholson et al., 1999; Zeil and Fleischmann, 2019; Vermehren et al., 2020), and it will be instructive in our model to investigate the effect of concentrating MB learning to key points of the route. Finally, we demonstrated our model could function in a real-world navigation context by embodying it on a robot and comparing it to standard vision-based navigation algorithms. Although parameters were not optimal for the routes the robot was tested on, and the processing time was slower than the standard visual navigation algorithms, it provides existence proof that the model can work in closed loop and is a solid foundation for future investigations focusing more on the active motor aspects of visual route navigation.

Due to the methodological limitation of neuroscience studies with ants, there is relatively little information on the exact information that reaches the mushroom body, but we can use information from other insects to theorise about this. In the bees (another insect in the Hymenoptera order) the lobula is known to be especially responsive to motion cues (Paulk and Gronenberg, 2008), so it is plausible that the MB may be able to make use of motion cues available to it in addition to the standard visual information from the optic lobe. In future it would benefit the field to see more MB modelling studies approach the challenge of incorporating temporal information into their simulations, as seen in (Zhu et al., 2021). There is evidence from ant behaviour that the sequence that ants learn visual stimulus affects their navigation routes (Schwarz et al., 2020), which has been explored in modelling more generally in relation to insect mushroom bodies (Arena et al., 2015). With respect to downstream projections of the mushroom body we can use data from *Drosophila* brain mapping and connectomics to suggest what regions the MBONs project to and speculate about potential implications of this connectivity. There is evidence that the *Drosophila* MB projects to the fan-shaped body and noduli in the central complex, and in a minor way to the lateral accessory lobe (LAL) (Li et al., 2020). The central complex plays a key role in integrating information from various sources to provide heading directions for navigation (Honkanen et al., 2019). The *Drosophila* central complex contains the ellipsoid body which encodes heading directions as bumps of activity with dynamics similar to that of a ring attractor network (Kim et al., 2017), and some predict that MB output could affect an animals heading direction by acting on this region and surrounding areas (Collett and Collett, 2018; Goulard et al., 2023).

While the LAL communicates information from the central complex to motor areas (Namiki and Kanzaki, 2016), meaning that the MB can exert influence on areas directly affecting the insect’s movement. MBONs also project to the neuropils of the protocerebrum (Aso et al., 2014), including the superior medial protocerebrum which is a relay to the LAL. While similar investigations have not yet been done in ants, if MB connectivity is somewhat similar, then this could be a plausible pathway from visual scene processing to movement.

Although we have some limited anatomical evidence and many theories based on other species of the connectivity of various regions to the ant mushroom body, the way visual memories are used and how they drive navigation is not yet known. We believe our model is therefore a great testbed for the various possibilities to see which are plausible. For instance, the approach we have demonstrated here is the standard/most simple navigation approach where the model scans many directions and travels in the direction with the highest familiarity score. Similar to (Ardin et al., 2016). However, it is unknown what the equivalent of scanning through all possible orientations of input would be biological. Ants do demonstrate scanning behaviours while navigating, however these scans involve physically rotating and although the oscillation patterns are constant (Clement et al., 2023) there are observable instances where ants will stop or increase scanning in a certain area (Wystrach et al., 2014). To overcome this potential scanning implementation problem, another approach would be to have left and right mushroom bodies working in parallel so that whichever side has the higher familiarity score causes a turn in the opposing direction as seen in (Wystrach, 2023; Steinbeck et al., 2024). When modelling this in a closed loop system, a lot of processing time would be saved from not having to scan through multiple orientations of images, but on the other hand there will be two entire mushroom bodies being simulated which may significantly increase the memory requirements for the running system. A contrasting approach involving a different method of training would be to class different views as attractive (towards the goal), and other views as repulsive (those facing away from the goal) as seen in (Möel and Wystrach, 2020). The benefits of this method would be the lack of scanning required, but it may struggle when faced with sinuous routes. Finally, one more way of potentially using visual memories would be to have the model be attracted around a “Ridge of familiarity” as seen in (Stankiewicz and Webb, 2021). In this example the virtual ant has a central pattern generator circuit causing repeated curved trajectories of movement as seen in (Clement et al., 2023), while a different process measures the changes in familiarity. The movement of the ant can therefore be modulated to oscillate around the area with the highest familiarity. As these examples demonstrate, there are many theories of how MB memories are utilised for navigation. Combining these theories with anatomical insight tested them in embodied models will likely provide new insight into the neural basis of ant navigation in the future.

## Data Availability

The datasets presented in this study can be found in online repositories. The names of the repository/repositories and accession number(s) can be found below: https://figshare.com/s/f8cb8d44479dfdbd1842 DOI: 10.25377/sussex.25118383 “Stanmer park outdoor navigational data”.

## Supplementary material

**Supplementary Video 1:**
Video showing a navigation trial of the mushroom body spiking neural network model running on a robot. Video is at 10x speed.
